# How to maintain the oral health of a child with Wolff-Parkinson-White syndrome: a case report

**DOI:** 10.1186/1752-1947-8-323

**Published:** 2014-09-30

**Authors:** Tsampikos Petroniatis, Eleonora Ortu, Nicola Marchili, Mario Giannoni, Giuseppe Marzo, Annalisa Monaco

**Affiliations:** 1 Unit of Dentistry, Department of Life, Health and Environmental Sciences; Division of Gnathology; Dental Clinic, University of L’Aquila, Via Vetoio, 67100 L’Aquila, Italy

**Keywords:** Wolff-Parkinson-White syndrome, Child, Oral health, Hygiene

## Abstract

**Introduction:**

Wolff-Parkinson-White syndrome is one of the most important disorders of the heart conduction system. It is caused by the presence of an abnormal accessory electrical conduction pathway between the atria and the ventricles.

**Case presentation:**

In the present report, we describe the correct oral health management of a 12-year-old Caucasian girl with Wolff-Parkinson-White syndrome.

**Conclusions:**

We successfully undertook the dental care of a girl with Wolff-Parkinson-White syndrome, which we describe here.

## Introduction

Wolff-Parkinson-White syndrome (WPWS) is a disease, described for the first time in 1930, characterized by a short PR interval associated with ventricular preexcitation manifested by a delta wave. This disease can generate symptomatic or asymptomatic arrhythmias and, in the most unfortunate cases, sudden death. Cardiac electrical activity starts in the sinus node, physiologically, located in the right atrium, propagates through the atrioventricular node and through the bundle of His into the ventricles. The atrioventricular node functions as a gate, limiting the electrical activity that reaches the ventricles. Patients with WPWS have an accessory pathway that connects the atria and ventricles, in addition to the atrioventricular node. This accessory pathway is the bundle of Kent. This accessory bundle can conduct electrical impulses much faster than atrioventricular node. This event itself is unfavorable: heart rates as fast as they occur in this disease, may develop hemodynamic problems and cardiovascular shock [1-3]. The diagnosis is made by electrocardiogram (ECG) in subjects without symptoms. Typical signs of the disease are: supraventricular tachycardia (38 percent), palpitations (22 percent), chest pain (5 percent), syncope (4 percent), atrial fibrillation (0.4 percent), sudden death (0.2 percent), and incidental findings (26 percent); data were unavailable in 4 percent. Subjects can also develop lightheadedness and/or dizziness [4,5]. The treatments of the pathology are drug therapy, radiofrequency ablation, and surgical ablation. The patients who suffer from atrial fibrillation and rapid ventricular response are treated with amiodarone or procainamide to monitor always their heart rate [6]. AV node blockers should be avoided in atrial fibrillation and atrial flutter with WPW or history of it; this includes adenosine, diltiazem, verapamil, other calcium channel blockers and beta-blockers may aggravate the syndrome by blocking the normal electrical pathway of the heart. The definitive treatment of WPW is a destruction of the abnormal electrical pathway catheter ablation radiofrequence [7]. It is very important to maintain the best oral health in these patients. There is no documented evidence in the literature. Patients with this type of pathology must be kept under control and must undergo specialized controls consistently. In addition, these patients should be able to maintain a good level of oral health. Dental procedures must be kept under antibiotic cover only when it is necessary and the use of equipment that bestow pulses of electrical stimulation (transcutaneous electrical nervous stimulation (TENS), radiofrequency scalpel, piezosurgery) is banned because it may interfere with heart rhythm [8]. Regarding procedures, such as dental calculus removal, pursued by mechanical equipment, clinicians are encouraged to ask for specialist advice from the cardiologist. The aim of this manuscript is to show how to behave in the case of WPWS in a child, in order to adopt best practices without interfering with the pathology.

## Case presentation

**Figure 1:**
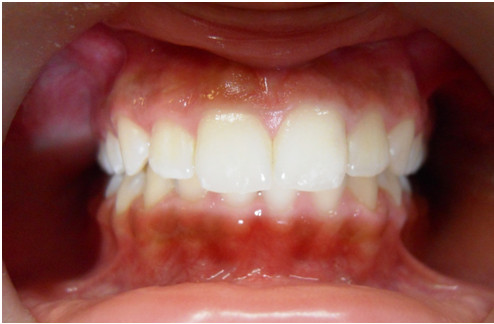
Frontal intraoral photo.

**Figure 2:**
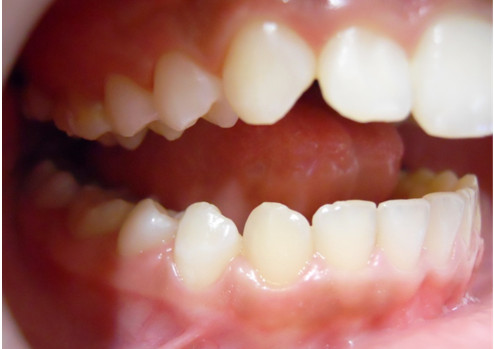
Buccal intraoral right photo.

**Figure 3:**
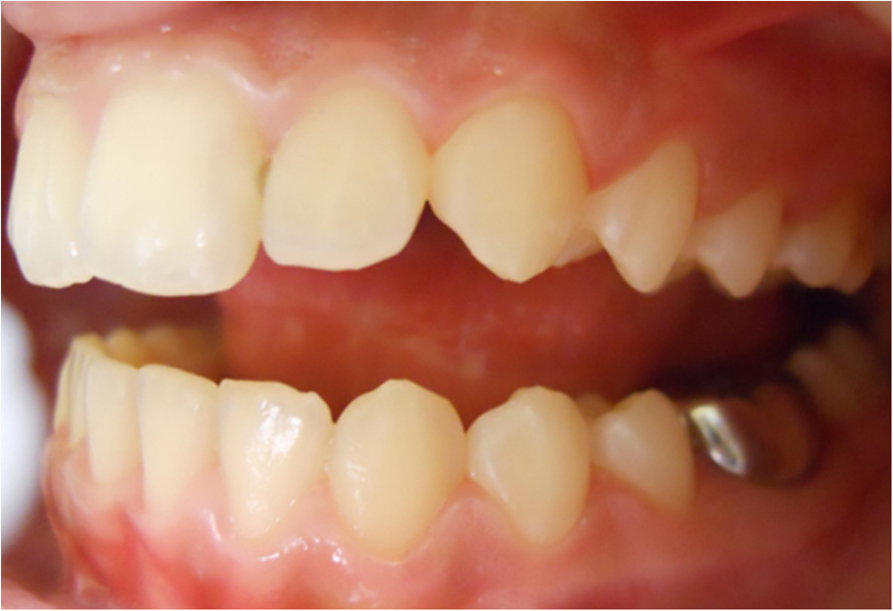
Buccal intraoral left photo.

**Figure 4:**
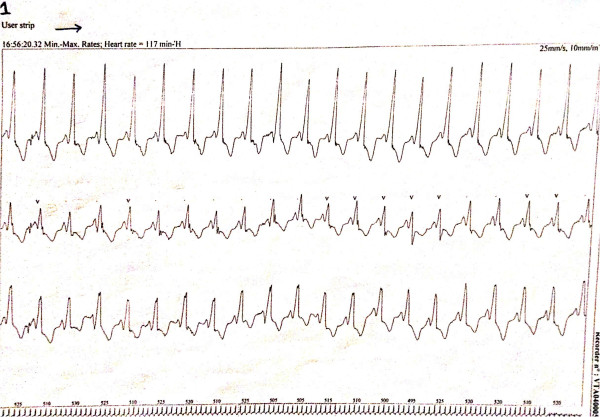
Electrocardiogram of the patient with Wolff-Parkinson-White syndrome.

**Figure 5:**
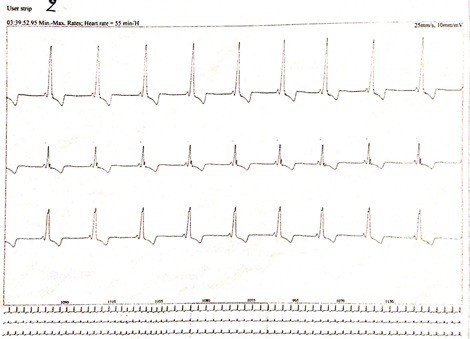
Electrocardiogram of the patient- note the characteristic delta wave, the PR interval, and the QRS complex.

**Figure 6:**
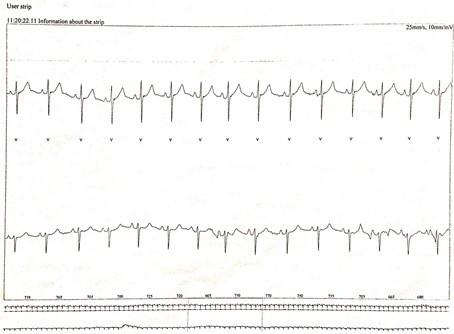
Electrocardiogram of the patient-sinus rhythm with alternating conduction through the atrioventricular node and the bundle side.

A 12-year-old Caucasian girl was presented to our dental clinic for a dental visit. An extraoral examination did not reveal facial asymmetry. Intraoral examination showed that her dental development was age-appropriate (Figures 1, 2 and 3). During the anamnestic interview, the parents of our patient referred to a light unintentional activity of rubbing of the teeth by the child during the night. Our patient was also under the care of a cardiologist for the presence of WPWS with frequent episodes of supraventricular tachycardia. The ECG (Figures 4, 5 and 6) performed one month before the dental visit showed the sinoatrial rhythm with alternate conduction medium atrioventricular node and the accessory conduction pathway. For this disease, our patient was treated with flecainide acetate (30mg three times a day) and propanolol 40mg once a day. Polysomnography was also performed on the child to evaluate the presence of pediatric obstructive sleep apnea (OSA) but the result was normal. Our patient was taken under the care of dentists and hygienists to achieve good oral health. In collaboration with the cardiologist, a treatment plan was created that included:

– periodic checks to be carried out every three months;

– sessions of oral hygiene with manual and mechanical instrumentation to be carried out at least every six months under antibiotic therapy;

– oral hygiene instructions and motivation;

– sealing of the first molars and fluoride use.

If more invasive treatments are needed in the future, they will be performed under antibiotic therapy and in collaboration with the cardiologist.

## Conclusions

The aim of this case report is to show how to maintain the correct oral health in a child with WPWS. There is no evidence of this problem in the scientific literature. The paroxysmal ventricular tachycardia can have different ways of presentation: WPWS is one of these. However, it is scientifically clear that the WPWS causes tachycardia, due to the disturbance of cardiac conduction. Tachycardia is an alteration of the autonomic nervous system (an increased tone of the sympathetic nervous system) [9]. The differential diagnosis with other diseases is more important. The ECG findings in people with the WPWS pattern can be similar to ECG findings seen in other cardiac conditions: myocardial infarction, ventricular premature beats or idioventricular rhythm, bundle branch block. Finally, some cases of hypertrophic cardiomyopathy may mimic WPWS [10]. Nowadays, one of the purposes to be achieved, by dentists and hygienists, is to keep the patient as relaxed as possible, explaining the procedures that will be performed each time to avoid unnecessary fears that can speed up the heartbeat. Several other issues are also discussed, such as the importance of continual collaboration with medical colleagues, the risk-benefit of using epinephrine-containing local anesthesia for dental treatment for patients with arrhythmias, the potential risk of repeated general anesthesia in a patient with a cardiac arrhythmia, and the challenges of providing comprehensive dental treatment in a high caries-risk patient with extreme dental anxiety [11]. Arrhythmias can be also induced by compression of the neck, the carotid sinus or eyes (vagal reflex). The risk is greater in older people and in patients with coronary artery disease or aortic stenosis. Arrhythmias may occur following administration of erythromycin or azole antifungal drugs in patients taking terfenadine, astemizole, or cisapride. Syncope may be the consequence of a bradycardia, a branch block or atrial tachycardia, and it may be recognized by the slowness or irregularity of the heartbeat. Such signs are important to distinguish syncope from a simple faint, although the immediate treatment is the same. Ventricular fibrillation is clinically indistinguishable from asystole and is one of the most serious emergencies that it may be necessary to treat in a dental clinic. Some antiarrhythmic drugs can cause oral lesions. Verapamil, enalapril and diltiazem can cause gingival hyperplasia; some beta blockers can, although rarely, cause lichenoid lesions; procainamide can cause lupus-like lesions [12]. If more invasive treatments are needed, the authors suggest to treat patients always under antibiotic therapy and in collaboration with the cardiologist. It is therefore very important to always check these patients even in collaboration with other medical specialists.

## Consent

Written informed consent was obtained from the patient’s parent for publication of this case report and any accompanying images. A copy of the written consent is available for review by the Editor-in-Chief of this journal.

## Abbreviations

ECG: electrocardiogram; OSA: obstructive sleep apnea; TENS: transcutaneous electrical nervous stimulation; WPWS: Wolff-Parkinson-White syndrome.

## Competing interests

The authors declare that they have no competing interests.

## Authors’ contributions

Our patient was under the care of AM and MG; AM and GM operated on our patient. EO analyzed and interpreted the data. EO, NM and TP wrote the manuscript. All authors reviewed and approved the final manuscript.
